# Thyroid and Heart: Severe Three Vessel Coronary Artery Disease in a Middle-Aged Female with Hypothyroidism

**DOI:** 10.7759/cureus.6095

**Published:** 2019-11-08

**Authors:** Ramsha Muneer, Syed Arsalan Ahmed Naqvi, Owais Gul, Syed Danish H Zaidi, Mudassir Iqbal Dar

**Affiliations:** 1 Internal Medicine, Dow University of Health Sciences, Karachi, PAK; 2 Cardiac Surgery, Civil Hospital Karachi, Karachi, PAK

**Keywords:** hypothyroidism, three vessel disease, cabg

## Abstract

Hypothyroidism is a common medical condition. The low metabolic state in hypothyroidism leads to significant cardiovascular and hemodynamic changes. Hypothyroidism is associated with heart failure, diastolic hypertension, atherosclerosis, coronary artery disease (CAD), and decreased insulin sensitivity. Similarly, the administration of levothyroxine worsens the cardiovascular disease by establishing a supply-demand mismatch. Here, we present a case of a 45-year-old woman with hypothyroidism who presented to us with exertional chest pain and later got diagnosed with severe three-vessel disease. Coronary artery bypass grafting (CABG) surgery was planned after the establishment of euthyroid state.

## Introduction

Thyroid hormones play a major role in regulating the metabolic physiology of the human body and exert significant effects on the cardiovascular system. Hypothyroidism is associated with various cardiometabolic changes. These changes include impaired diastolic function, decreased cardiac contractility, endothelial dysfunction, decreased endothelial nitric oxide production, decreased arterial compliance, and decreased vascular smooth muscle relaxation. This low metabolic state in hypothyroidism leads to heart failure, diastolic hypertension, atherosclerosis, and sometimes coronary artery disease (CAD) [1]. Similarly, decreased insulin sensitivity due to the downregulation of glucose transporters, insulin resistance, or decreased insulin secretion can also be associated with hypothyroidism [2]. We present a case of a 45-year-old woman having a three-vessel disease with underlying severe hypothyroidism, non-insulin dependent diabetes mellitus, and hypertension, who presented to us with typical chest pain.

## Case presentation

A 45-year-old married woman presented to the emergency department with the primary complaints of chest pain and palpitations for a week. She was diagnosed with hypothyroidism five years back for which she had been on thyroid replacement therapy. However, she was diagnosed with hypertension and diabetes mellitus a couple of years back. She reported that she had been experiencing chest pain while performing household activities accompanied by severe shortness of breath, paroxysmal nocturnal dyspnea, and occasional episodes of nausea. The pain was sudden in onset, throbbing in character, radiating to both arms, and was relieved by sublingual nitroglycerin. She denied any symptoms of accompanying cough, syncope, joint pain, or edema. Furthermore, her history ruled out any previous trauma and symptoms related to congenital heart disease, congestive cardiac failure, or myocardial infarction. Her past medical and surgical history was insignificant. Her systemic review, which was otherwise unremarkable, however, indicated hair loss, significant weight gain, and infrequent bowel habits. There was no history of tobacco, alcohol, or recreational drug use. Her family history was insignificant for cardiovascular disorders or autoimmune disorders of any sort. She had been taking losartan 25 mg/day and oral hypoglycemic drugs for hypertension and diabetes mellitus, respectively.

On physical examination, she was fully alert and oriented. The patient was afebrile with a regular pulse rate of 75 beats per minute (BPM) and a blood pressure of 110/70 millimeter of mercury (mmHg). Jaundice, lymphadenopathy, cyanosis, and edema were not found. Neck examination was unremarkable except for a mildly enlarged thyroid. Cardiac examination did not reveal any abnormal heart sounds or raised jugular venous pulse. Lungs were clear to auscultation bilaterally with a respiratory rate of 20 breaths per minute. The abdominal and neurological examinations were insignificant. Electrocardiography (ECG) showed sinus rhythm. The patient was admitted for further evaluation.

Complete blood count (CBC), erythrocyte sedimentation rate (ESR), C reactive protein (CRP), coagulation profile, liver function tests (LFTs), blood urea nitrogen (BUN), creatinine (Cr), and basic metabolic panel (BMP) were within normal limits. Cardiac Troponin-I (cTnI) and thyroid stimulating hormone (TSH) were elevated with values of 5.19 nanograms/milliliters (ng/ml) and 70 milli international units per liter (mIU/L), respectively. Similarly, HbA1c was also found elevated at 8.8%.

**Figure 1:**
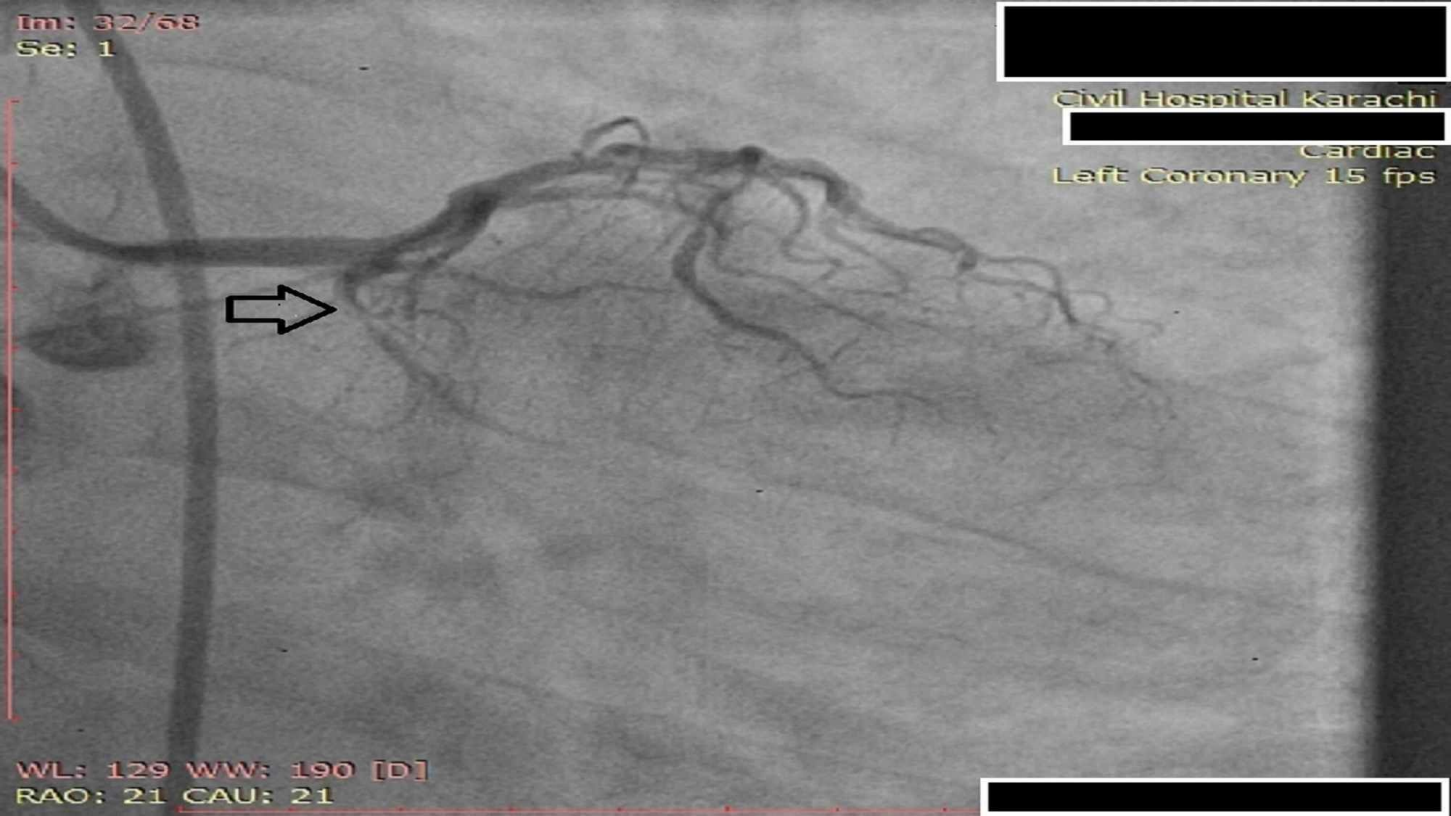
Angiogram of LCX coronary artery showing severe (90-100%) distal segment stenosis LCX: Left circumflex

**Figure 2:**
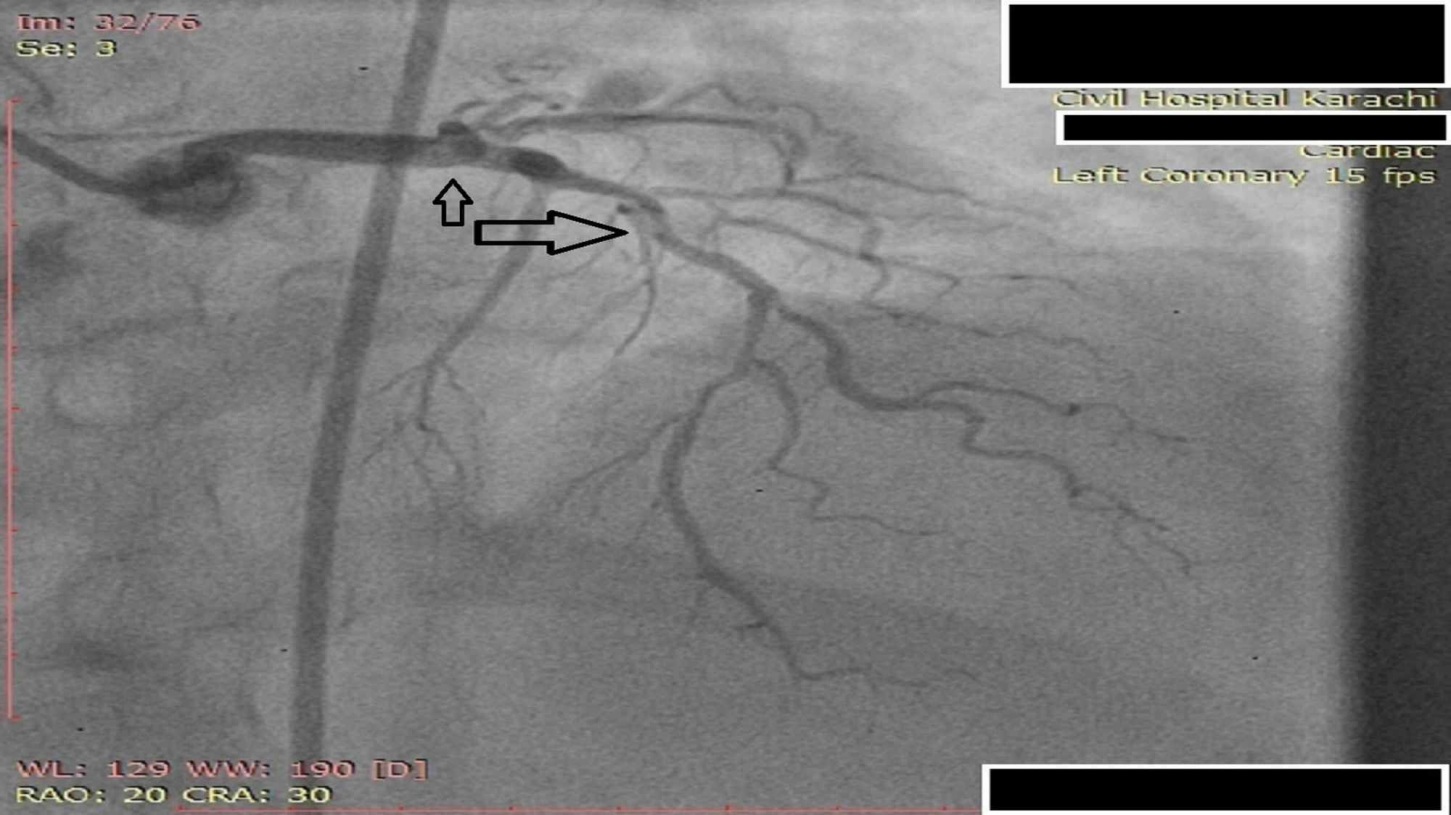
Angiogram of LAD coronary artery showing moderate (50%) proximal segment and severe (70%) mid-segment stenosis LAD: Left anterior descending

**Figure 3:**
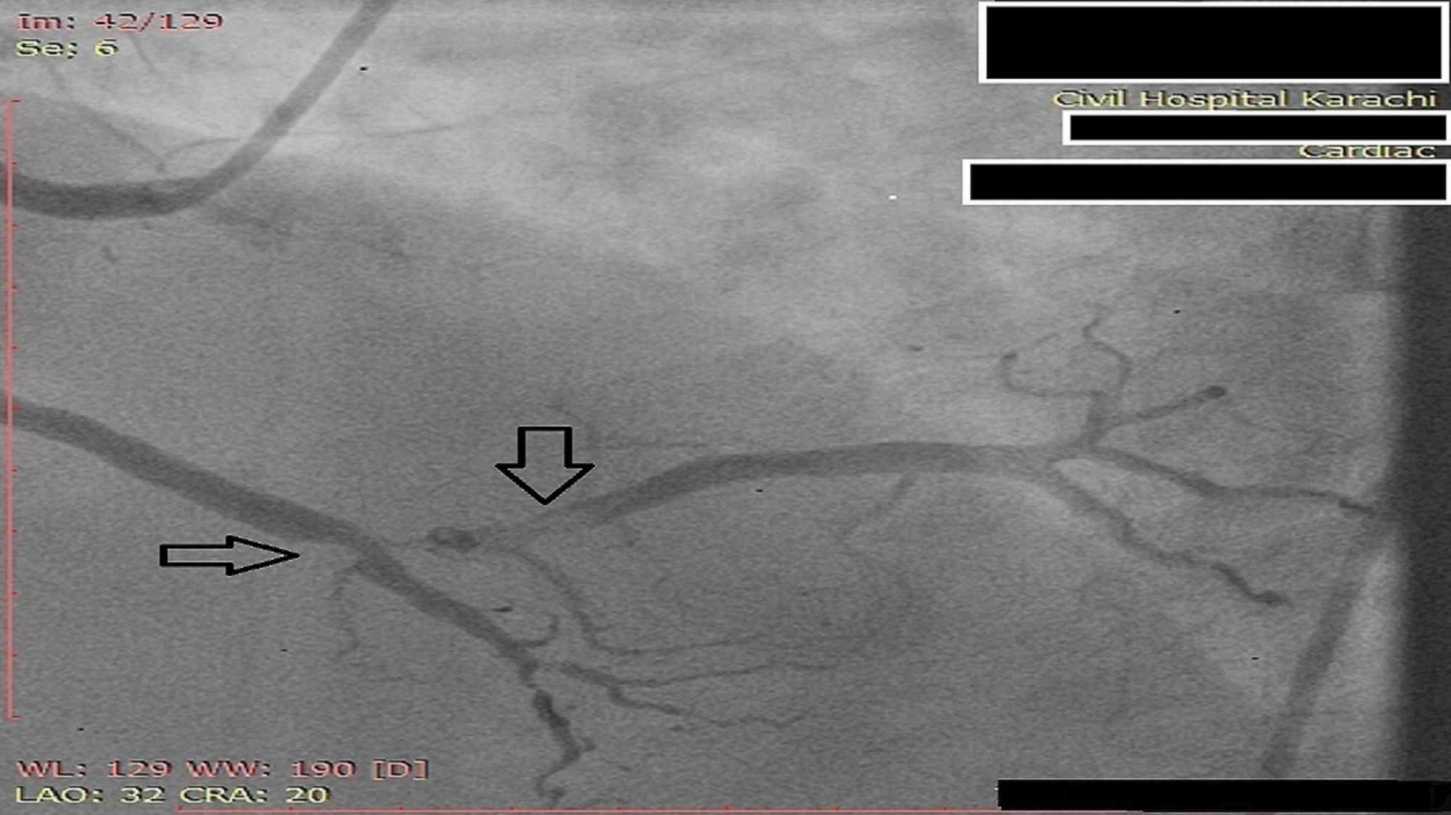
Angiogram of RCA showing severe (80-90%) blockage with complete stenosis of PDA RCA: Right coronary artery, PDA: Posterior descending artery

Non-invasive imaging tests were performed, which included echocardiography and neck ultrasound. The echocardiography revealed segmental posterior wall motion abnormality and grade one diastolic dysfunction. The neck ultrasound did not reveal any significant findings. Her thyroid scan was normal. Later, cardiac angiography was done, which showed severe three-vessel disease in a right dominant heart circulation (Figures 1-3).

Coronary artery bypass grafting (CABG) surgery was planned after the establishment of euthyroid state. She was started on multiple drugs, which included aspirin 75 mg/day, metoprolol 25 mg twice a day, ranitidine 5 mg/day, thyroxine 500 mg/day, and gliclazide 30 mg twice a day. The patient was advised to return for surgery after a week; however, she did not come back.

## Discussion

Hypothyroidism is a commonly encountered clinical condition that affects between 4-10% of the population [1]. Overt hypothyroidism is diagnosed when low levels of the thyroid hormones result in elevated levels of thyroid-stimulating hormone (TSH) above 4.0 mU/L whereas, subclinical hypothyroidism is diagnosed when TSH levels are elevated above the upper limit of the assay reference range with normal thyroid hormone levels. Thyroid hormones play a paramount role in the normal function of the heart and vascular physiology. Thus, hypothyroidism produces significant cardiovascular effects. Hypothyroidism is known to affect cardiac contractility, which is often diastolic in nature and can lead to decreased cardiac output. Similarly, increased systemic vascular resistance, decreased arterial compliance, and atherosclerosis are common pathophysiological changes that occur in hypothyroidism [3,4]. Although thyroid hormone deficiency, either clinical or subclinical, is an established risk factor for cardiovascular disease, it is unusual to find three-vessel coronary artery disease in a middle-aged woman in our setup who had an insignificant family history and no long-standing coronary artery disease risk factors.

 On review of the literature, a case to similar ours was reported from Japan, where a 38-year-old woman with hypothyroidism presented with unstable angina and subsequently underwent CABG [5]. Another case was reported from China, where a patient with hypothyroidism, type 2 diabetes mellitus, and hypertension underwent CABG [6]. Similar cases have been reported from Asia, involving men and women both [7,8]. Many studies conducted across the globe revealed a significant association between subclinical hypothyroidism and subsequent development of ischemic heart disease (IHD) [9-11]. A 2017 meta-analysis of 55 cohort studies concluded that hypothyroidism is associated with higher risks of IHD, cardiac mortality, and all-cause mortality when compared with euthyroidism in the general public or in patients with preexisting cardiac disease. It also noted that subclinical hypothyroidism with elevated TSH levels is associated with an increased risk of IHD events, especially in those with values greater than 10 mIU/L, similar to our case [12]. The findings of these studies show that even subclinical hypothyroidism is a risk factor for coronary artery disease, not alone overt hypothyroidism. In contrast, the European Prospective Investigation into Cancer and Nutrition (EPIC)‐Norfolk study did not show any increased risk of coronary artery disease [13].

Moreover, thyroxine replacement therapy is considered as the mainstay treatment for overt hypothyroidism. The use of thyroid replacement therapy can accelerate the development of ischemic heart disease or aggravate the progression of an already existing cardiac disease. This could be due to increased cardiac contractility and improved metabolism, which eventually leads to oxygen supply-demand mismatch resulting in myocardial ischemia and possibly myocardial infarction. One such case was reported in Japan, where the administration of levothyroxine aggravated the patient’s clinical condition [5]. Similarly, Flynn et al. reported a population-based study of patients with elevated TSH levels (defined as greater than 4 mIU/L) and treated with levothyroxine presented with greater risk for cardiovascular events [14]. In another study, no evidence was found to suggest clinically meaningful differences in the pattern of long term health outcomes including all-cause mortality, heart failure, IHD, stroke/transient ischemic attack, and atrial fibrillation in patients on thyroid replacement therapy when TSH concentrations were within normal limits and thus, further emphasizing the need of randomized controlled trials of levothyroxine treatment examining vascular and cardiovascular outcomes [15]. Hence, it is crucial to conduct large scale studies aimed at investigating possible risk factors including thyroid replacement therapy that lead to the development of coronary artery disease in patients with subclinical or overt hypothyroidism, especially in our locality where there is a scarcity of data available on this subject.

## Conclusions

Thyroid hormones play an important role in regulating the heart and vascular physiology. Hypothyroidism, either overt or subclinical, is associated with significant cardiovascular effects. Three vessel disease is a relatively uncommon cardiovascular manifestation of hypothyroidism as most patients present with diastolic dysfunction. Thyroid replacement therapy can accelerate the development of cardiovascular disease or aggravate the underlying heart conditions according to some reports. Therefore, more studies should be conducted to investigate and evaluate the risk factors in hypothyroid patients that can lead to the development of coronary artery disease.
